# Performance verification of the new fully automated Aquios flow cytometer PanLeucogate (PLG) platform for CD4-T-lymphocyte enumeration in South Africa

**DOI:** 10.1371/journal.pone.0187456

**Published:** 2017-11-03

**Authors:** Lindi-Marie Coetzee, Deborah K. Glencross

**Affiliations:** 1 Department of Molecular Medicine and Haematology, Faculty of Health Sciences, University of the Witwatersrand, Johannesburg, South Africa; 2 National Health Laboratory Service (NHLS), CD4 Unit, Charlotte Maxeke Hospital, Johannesburg, South Africa; Central University of Tamil Nadu, INDIA

## Abstract

**Background:**

The National Health Laboratory Service (NHLS) offers wide-scale CD4 testing through a network of laboratories in South Africa. A new “load and go” cytometer (Aquios CL, Beckman Coulter), developed with a PLG protocol, was validated against the predicate PLG method on the Beckman Coulter FC500 MPL/CellMek platform.

**Methods:**

Remnant routine EDTA blood CD4 reference results were compared to results from two Aquios/PLG instruments (n = 205) and a further n = 1885 samples tested to assess daily testing capacity. Reproducibility was assessed using Immunotrol^TM^ and patient samples with low, medium, high CD4 counts. Data was analyzed using GraphPad software for general statistics and Bland-Altman (BA) analyses. The percentage similarity (%Sim) was used to measure the level of agreement (accuracy) of the new platform versus the predicate and variance (%SimCV) reported to indicate precision of difference to predicate.

**Results:**

205 samples were tested with a CD4 count range of 2–1228 cells/μl (median 365cells/μl). BA analysis revealed an overall -40.5±44.0cells/μl bias (LOA of 126.8 to 45.8cells/μl) and %Sim showing good agreement and tight precision to predicate results (94.83±5.39% with %SimCV = 5.69%). Workflow analysis (n = 1885) showed similar outcomes 94.9±8.9% (CV of 9.4%) and 120 samples/day capacity. Excellent intra-instrument reproducibility was noted (%Sim 98.7±2.8% and %SimCV of 2.8%). 5-day reproducibility using internal quality control material (Immunotrol™) showed tight precision (reported %CV of 4.69 and 7.62 for Normal and Low material respectively) and instrument stability.

**Conclusion:**

The Aquios/PLG CD4 testing platform showed clinically acceptable result reporting to existing predicate results, with good system stability and reproducibility with a slight negative but precise bias. This system can replace the faded XL cytometers in low- to medium volume CD4 testing laboratories, using the standardized testing protocol, with better staff utilization especially where technical skills are lacking. Central monitoring of on-board quality assessment data facilitates proactive maintenance and networked instrument performance monitoring.

## Introduction

Flow Cytometry (FC) has been the testing platform of choice for CD4 T-lymphocyte enumeration in HIV infected patients [1–3] since the correlation between CD4 loss and HIV disease progression was first described [4–6]. South Africa has a high burden of disease with 11.2% (6.2 million) of the local populace living with HIV in 2015 (Statistics South Africa, 2015) and an average of ~ 9.7% of HIV+ patients noted to have CD4 counts less than 100cells/μl [7].

With the onset of the national HIV program in South Africa and the development of strategic HIV and AIDS Plans for South Africa [8–10], the challenges of providing CD4 enumeration as a routine test across multiple laboratories, led to the local development of a low-cost, easy-to-use, single platform, 2-color, lyse-no-wash cytometry assay, i.e. the PanLeucogate (PLG) CD4 [11–13]. This protocol incorporated CD45 FITC and CD4 PE labeled antibodies with a gating strategy based on total white cells as reference rather than using the total lymphocyte population as reference as recommended previously [14, 15]. With PLG, white cell separation is based on the differential expression of CD45 and CD4, without the need for costly additional antibodies to define T-cells (CD3) or exclude monocytes (CD14), B-lymphocytes (CD19) and natural killer cells (CD16/56) in the CD4 gating strategy. This PLG protocol was implemented onto Beckman Coulter cytometers, i.e. the XL and later the fully automated FC500 MPL/CellMek system, which through a national procurement tender, became the predicate CD4 testing platform for the National Health Laboratory Service (NHLS). By the end of 2015, 18 laboratories were using XL instruments (n = 18 systems) while 34 laboratories used MPL/CellMek (n = 66 systems) as single or multiple test platforms across 52 CD4 testing laboratories.

Ongoing development of the PLG/CD4 assay has seen the introduction of sample-by-sample quality assessment through monitoring of the flow count rate (FCR), for each sample tested [16–18]. This and other quality assessment tools developed [12, 19, 20], including an external quality assessment scheme, [21] is now an integral part of the standardized CD4 assay adhered to across the network of testing facilities (n = 49 in 2017). The PLG/CD4 assay has also been instrumental as a base for extended HIV-related biomarker assay development such as CD38 on activated CD8 T-cells [22, 23] and a Cryptococcal antigen flow assay (in development) for testing patients with a CD4 count <100cells/μl [24, 25].

**Fig 1:**
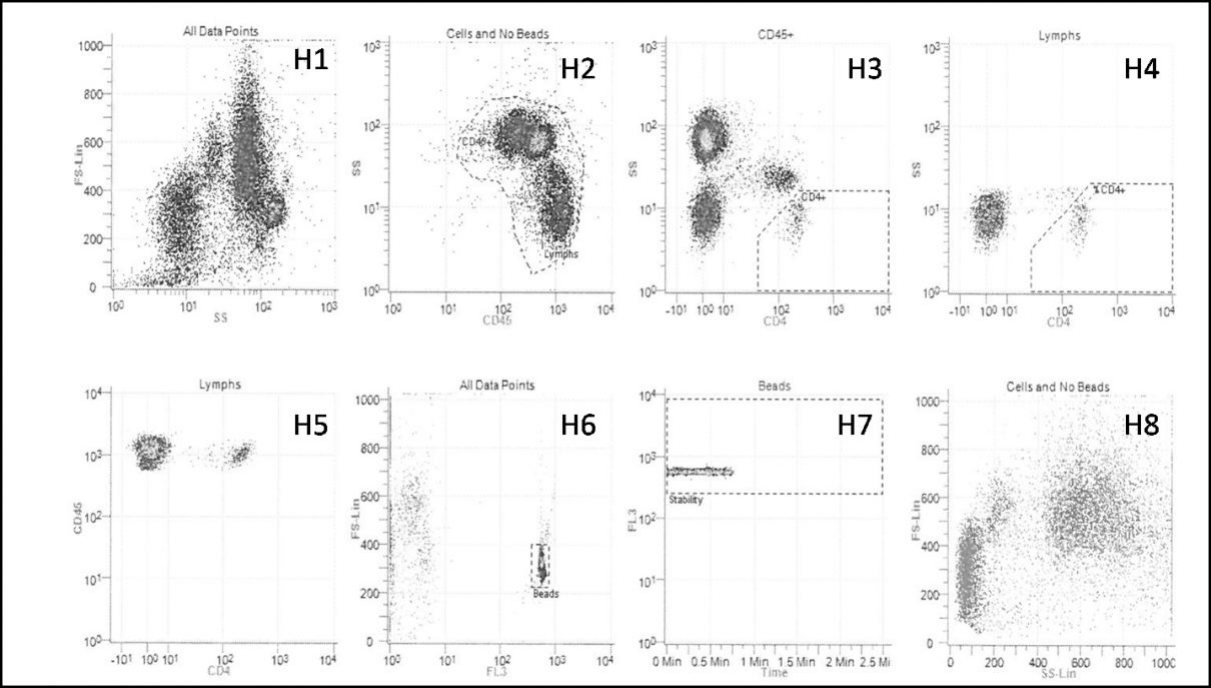
PLG/CD4 protocol on Aquios CL cytometer. PLG/CD4 gating strategy applied for the Aquios CL cytometer platform, represented by 8 histograms (H1-8). H2 selects all CD45 positive white cells, from which all CD4 bright lymphocytes are gated (in H3) for reporting of an absolute CD4 count, while a CD4% of lymphocytes value is derived from H4. H6 and H7 indicate the bead population, currently used as an internal control measure of instrument stability.

Beckman Coulter (Miami, FL) developed an operator independent CD4 testing platform, the Aquios CL cytometer as a small footprint, “load and go” true volumetric platform, requiring minimal flow cytometry skills/experience. The PLG protocol for the Aquios CL cytometer was specifically developed in collaboration with the CD4 reference laboratory in Johannesburg, to comply with the specifications of the NHLS predicate PLG/CD4 protocol as well as the internal quality assessment measures developed over time; i.e. FCR monitoring with Beckman Coulter Flow Count Beads^TM^ for within-sample monitoring of instrument stability and CD4 enumeration accuracy (Fig 1). Additional internal quality parameters i.e. mean channel value monitoring of CD45 vs. Side Scatter expression of white cell subsets were developed specifically for the Aquios PLG version and incorporated into the quality control module to monitor instrument stability and reproducibility of cell separation on Levy-Jennings plots.

This paper describes the verification study conducted as part of the NHLS internal requirements for evaluation of new technology through the Health Technology Assessment Unit (HTA) in accordance with international guidelines [26]. Direct comparison of the Aquios CL instrument performance against the predicate method in the NHLS (PLG/CD4 on FC500 MPL/CellMek system) was done to assess accuracy, precision and reproducibility of this new platform.

## Methods

### Verification procedure

The service provider met requirements for Installation Qualification (IQ), i.e. verification that equipment was installed, activated and put into use according to manufacturer’s requirements. After completion, Operator Qualification (OQ) was done as training of end users to operate the system, do daily quality assessments and maintenance as per manufacturer’s instructions. Performance qualification (PQ) followed to verify reproducibility, accuracy and precision of the testing platform against the predicate test platform/methodology (local reference method) prior to introducing the platform to the NHLS CD4 testing network as a replacement of fading XL cytometers. The outcome of the PQ verification is reported here with reference data available (S1 and S2 Tables and S1 File).

### Instruments and test samples

Two Aquios^TM^ flow cytometers with a manufacturer-installed PanLeucoGate (PLG) protocol were used for this study. The closed-system Aquios platform incorporates sample preparation, analysis and reporting, using a lyse-no-wash preparation method, with cell interrogation by a 488nm diode laser, 2 light scatter channels and an electronic volume (EV) measure. All reagents, including VersaFix Lysing reagent, PLG antibody panel (CD45 FITC/CD4 PE), Immunotrol^TM^ normal and low, Flow Count Beads and sheath fluid were provided by Beckman Coulter SA.

Remnant laboratory CD4 EDTA samples were used after testing with the predicate method, i.e. Beckman Coulter FC500 MPL/CellMek system with PLG protocol. All testing was done per manufacturer specifications, NHLS internal standard operating procedures (SOP’s) and laboratory safety regulations. Ethics clearance for the study was obtained through the University of the Witwatersrand (M12020 and M0909516).

### Performance Qualification (PQ)

#### Direct comparison with predicate platform/method

Accuracy of Aquios result reporting was initially assessed on 205 patient samples tested on the predicate PLG/CD4 F500 MPL/CellMek system and retested on the Aquios/PLG system on remnant EDTA blood within 24 hours of initial routine testing. An additional 1885 samples were also tested across three Aquios/PLG platforms to assess daily throughput, workflow and accurate result reporting under typical high volume testing laboratory conditions for ten consecutive days. Absolute CD4 count (#CD4) and CD4 percentage of lymphocytes (CD4%) were reported for all aspects of the PQ assessment.

#### Daily internal quality control (IQC) for instrument performance

The daily quality control measures for operating the Aquios system, includes analysis of Immunotrol^TM^ at both Normal and Low levels (Beckman Coulter, Miami, FL), with both #CD4 and CD4% values performed as per prescribed procedures by the manufacturer. This included analysis of Flow Check Fluorospheres (Beckman Coulter, Miami, FL) to monitor laser alignment, fluidics and electronic stability. Automated compensation was done when analyzing Immunotrol™ control material, with a ‘pass’ or ‘fail’ reported on the results. Immunotrol^TM^ Normal and Low were analyzed daily at startup and these results were captured for 12 consecutive days to assess accuracy (comparison to package insert values) and instrument performance precision over time. Additional daily IQC measures included background count and carry-over and a weekly reproducibility to comply with current CD4 enumeration practices in the NHLS (S1 File).

#### Reproducibility

Reproducibility was assessed using Immunotrol^TM^ at 2 Normal and 2 Low levels (2 batches of control material), where the same Immunotrol™ tube was tested ten consecutive times through the single tube loader. In addition to manufacturer controls, reproducibility was also done on random patient samples with confirmed low (<100cells/μl), medium (300–400 cells/μl) and high (>500 cells/μl) CD4 counts, by analyzing each sample ten consecutive times.

#### External quality control (EQA) performance assessment

EQA material (samples) was obtained from the NHLS CD4 Proficiency Testing Scheme (PTS) external quality assessment program [12, 21] as a discrete panel of 10 consecutive retrospective scheme samples with known outcomes. These samples were tested on the Aquios and the results converted to a standard deviation index (SDI) value, using the available reported trimmed global mean and standard deviation of the retrospective trials. The SDI value was used as an indication of performance against a pool of 850 participants from 26 countries, using 8 different testing platforms [21]. Acceptable performance is an SDI value between -2 to 2 with an ideal target of zero.

#### Statistical analysis

Statistical analysis was done using GraphPad Prism6 Software, reporting basic statistics including minimum, maximum, median and mean values where indicated. 95% confidence intervals (95% CI) and coefficient of variation (%CV) was also calculated. In addition, the %Similarity model [27] was used to assess overall agreement between instruments or between the Aquios platform vs. the predicate PLG/CD4 on MPL/CellMek platform, with a mean, standard deviation and %CV (%SimCV) reported. As the % similarity model “over-estimates” the differences expressed as percentage of the reference value in samples with small numerical values, all samples with a CD4 count <50cells/μl were corrected to 100% similarity if the results reported by the Aquios and predicate systems were both less than <50cells/μl with the anticipated same clinical outcome, i.e. did not change patient treatment. Direct comparison of Aquios vs. MPL/CellMek results were analyzed with Deming regression (this analysis was performed for the entire data set of 205) and Bland-Altman statistics, where the bias with 95% Limit of Agreement (95% LOA) was reported for all samples as well as categories of absolute CD4 counts including <100 cells/μl (cut-off for identifying patients eligible for Cryptococcal antigen screening), <350/μl or <500cells/μl (based on 2014 and 2016 guideline cut-offs for therapy initiation) [9, 10, 28, 29]. Categorized data was also used to assess the number of miss-classified samples by the Aquios platform at the 100, 350 and 500cells/μl cut-offs.

## Results

### Direct comparison of samples tested on PLG/Aquios vs. MPL/CellMek PLG platform

In total, 205 samples were tested with an absolute CD4 count ranging from 2–1228 cells/μl (Table 1).

**Table 1:** Summary of samples tested using the predicate FC500 MPL/CellMek PLG platform and comparative statistics against the Aquios PLG platform.

**Absolute CD4 count**	**ALL**	**CD4≤100**	**Total CD4 0–350**	**Total CD4 0–500**	**CD4 >500**
**Number (% of total samples tested)**	205	47 (22.9%)	97 (47.3%)	149 (72.6%)	56 (27.3%)
**Mean (Range)**	365 (2–1228)	47 (2–99)	144 (2–348)	244 (2–497)	685.6 (504–1228)
**%Similarity (all samples)**	93.7±9.86	93.1±19.01	93.25±13.65	93.45±11.31	95.34±3.7
**%Similarity %CV (all samples)**	10.5	20.42	14.64	12.1	3.95
%Similarity (corrected*)	94.83±5.39	96.67±7.37	95.08±6.51	94.64±5.8	NA
%Similarity %CV (corrected*)	5.69	7.61	6.85	6.22	NA
**Bland-Altman Bias**	-40.5±44.0	-7.32±10.9	-18.98±22.3	-30.93±33.5	-66.04±57.4
**Bland Altman Bias (95%LOA)**	-126.8 to 45.8	-28.7 to 14.06	-62.58 to 24.62	-96.58 to 34.07	-178 to 45.96
**CD4% of Lymphocytes**	**ALL**	**CD4≤100**	**Total CD4 ≤350**	**Total CD4 ≤500**	**CD4 >500**
**Mean (Range)**	19.93 (0.67–50.24)	5.95 (0.67–14.38)	11.67 (0.57–35.32)	16.23 (0.67–50.24)	29.7 (14.7–49.6)
**%Similarity (all samples)**	98.32±8.13	93.36±14.93	96.33±11.21	97.4±9.32	100.8±1.88
**%Similarity %CV (all samples)**	8.27	15.99	11.63	9.57	1.87
%Similarity (corrected*)	99.45±4.03	98.29±5.71	98.72±5.11	98.9±4.49	NA
%Similarity %CV (corrected*)	4.06	5.81	5.19	4.54	NA
**Bland-Altman Bias**	-0.12±1.45	-0.74±1.72	0.42±1.49	-0.32±1.51	0.42±1.14
**Bland Altman Bias (95%LOA)**	-2.96 to 2.72	-4.12 to 2.63	-3.34 to 2.49	-3.27 to 2.63	-1.81 to 2.65

*Corrected indicates correction of %similarity to 100% for samples with a count of <50cells/μl (n = 27). See text for details.

**Fig 2:**
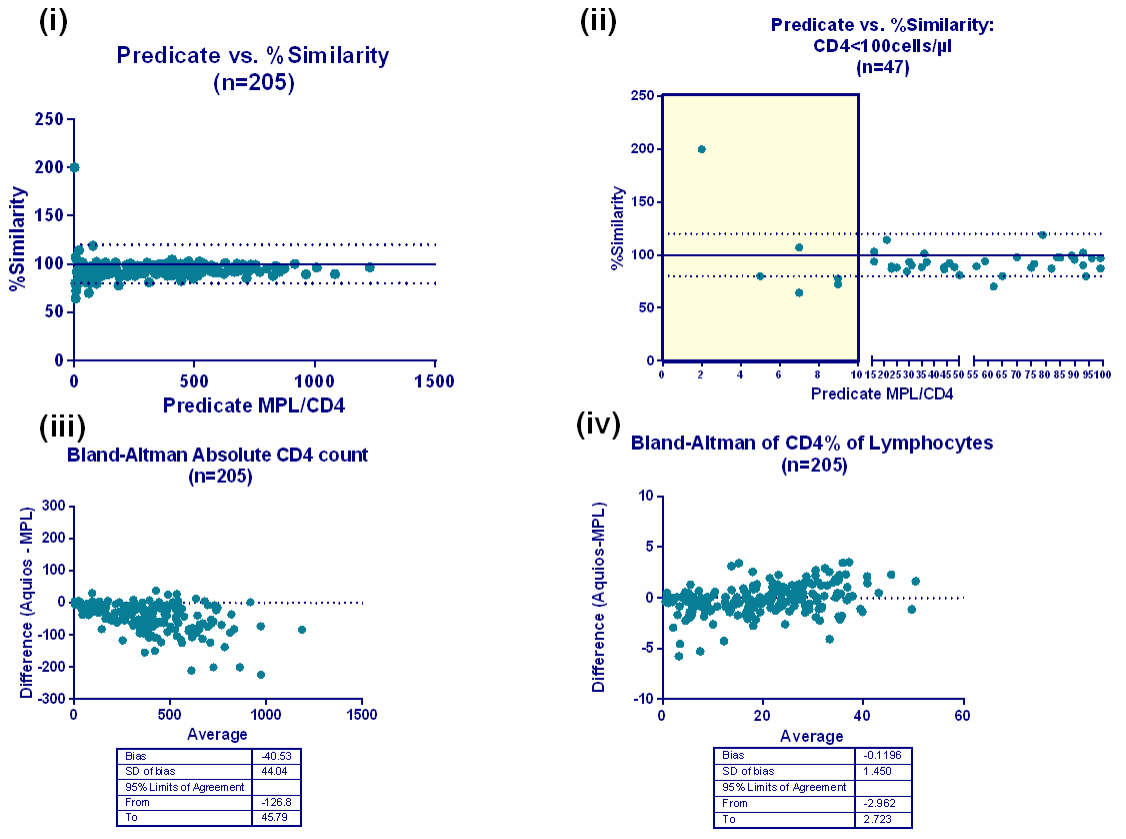
%Similarity and Bland-Altman analyses. (i) The % similarity (of Aquios vs. Predicate) plotted against the predicate absolute CD4 count indicating the distribution around the acceptable limits between 90–110%. (ii) A sub-analysis of CD4<100cells/μl of %Similarity vs. predicate absolute CD4 count reveals the greatest variability seen in samples with a numeric value <10 (shaded area). One marked outlier (arrow) reveals matching comparative counts of 2 cells/μl (predicate) vs. 4 cells/μl (Aquios). This example is used to provide evidence for the need to correct counts that are less than 50 cells/ μl to 100% similar and to avoid falsely biasing %Sim analysis outcomes. Below lie the Bland-Altman analyses (n = 205) comparing CD4 test platforms for (iii) absolute CD4 counts and (iv) CD4% of lymphocytes. Differences are expressed as Aquios (test) value minus MPL (predicate) value in the Bland Altman analyses.

Direct comparison against the predicate PLG/CD4 on FC500 MPL/CellMek platforms using the %Similarity model indicated an overall under-estimation of #CD4 by ~6% and per category of #CD4 counts. Fig 2 shows the relationship between the %Similarity and the #CD4 counts obtained with the predicate method, (i) for all samples and (ii) for samples with a CD4 count <100cells/μl. The latter shows the impact of samples with low numeric values on the overall % similarity calculation, hence the correction for comparative samples with counts <50cells/μl. These corrections did not affect the clinical outcome of patients as their CD4 counts remained <100 cells/μl.

An overall %Similarity for #CD4 of 94.83%±5.4 was noted with a %SimCV of 5.7%, revealing excellent precision of Aquios reporting in comparison to predicate results. Slightly higher, but acceptable variability, was noted in the #CD4 <100cells/μl category (%SimCV of 7.61%). Differences between platforms for CD4% of lymphocytes were only statistically significant in the# CD4<100cells/μl category, with overall good agreement with corresponding %SimCV values of <6% (Table 1).

Although a relatively constant difference of ~6% was evident from the %Similarity analysis, differences noted between absolute counts, as evidenced by the Bland Altman analysis (Fig 2iii and 2iv), were not clinically significant.

Misclassification rates at 100, 350 and 500 cells/μl levels revealed higher downward misclassification, confirming the under-estimation of absolute CD4 counts (calculated as number of incorrectly identified Aquios results higher than the threshold/number of reference values below the threshold x100). Upward misclassification (calculated as the number of incorrectly identified Aquios results that were lower than the threshold divided by the number of reference values above the threshold and multiplied by 100) at all thresholds were negligible at <2% (Table 2).

**Table 2:** Summary of misclassification of absolute CD4 counts at three thresholds of importance in HIV management, indicating the number of samples misclassified and the percentage of misclassification per threshold.

	Misclassification (n =, %)	
Threshold (cells/μl)	Upward	Downward
0–100	1 (2.13)	4 (2.53)
0–350	NONE	15 (13.9)
0–500	1 (0.7)	7 (12.5)

Bland-Altman analyses also confirmed the slight, though consistent under-estimation of #CD4 counts (Table 1 and Fig 2iii and 2iv) noted in the %Similarity analysis. An overall bias for #CD4 counts of -40.5cells/μl was observed, with a statistically and clinically insignificant bias for CD4% of -0.12%. Deming regression analysis of all #CD4 counts confirmed a good correlation between the testing platforms across a wide range of #CD4 counts (slope of 0.912±0.01; p,0.001 significant).

### Intra-platform comparison

A subset of samples (n = 50) were tested across two Aquios platforms for comparison to the predicate and one another. The %Similarity of instrument 1 vs. MPL and instrument 2 vs. MPL for #CD4 counts were 96.6±3.9% and 95.5±3.9% respectively with corresponding %SimCV values of <3.8%. The %Similarity for inter-instrument comparison was 98.7±2.8% with a %CV of 2.8% for #CD4 counts noted. Bland-Altman analysis confirmed a negative bias between 30 to 44 cells/μl compared to the predicate, with a very small bias of 12.9±27 (95% LOA of -66 to 40) for intra-instrument comparison for #CD4 counts.

### Workflow and capacity

All routine predicate CD4 tests were repeated and re-tested on three Aquios/PLG instruments over 10 consecutive working days (n = 1885) to evaluate workload capacity in the context of high workload volumes. This evaluation confirmed that each Aquios instrument can process between 95–115 samples per 8-hour day (including staff breaks during the day of ~total 75 minutes), comparing well with the capacity of the redundant XL flow cytometers of 120 samples per 8-hour day.

Bland Altman analysis of this data set confirmed the slight under-estimation of #CD4 counts and a bias of ˜45 cells/μl. Overall %Similarity analysis for #CD4 was 94.5±11.53% with a %SimCV of 12.2% and 99.4±8.3 (%SimCV of 9.4%) for CD4% values. Data points with matched values <50cells/μl (n = 138) corrected to 100% similar did not affect the overall clinical outcomes. Here the %Similarity for #CD4 changed only marginally to 94.9±8.9% (%SimCV of 9.4%) and 99.7±5.5% (%SimCV of 5.6%) for CD4%. An overall bias of -47.8 cells was reported (95% LOA from -211 to 116 cells/μl) (S2 Table).

### Daily quality control (DQC)

Immunotrol^TM^ Normal and Low were analyzed for 12 consecutive days (Table 3). Results were within package insert expected target ranges, with corresponding %CV values of <8% over the 12 days for both parameters at both Immunotrol™ levels.

**Table 3:** Summary of results from manufacturer provided daily quality control material (Immunotrol^TM^ Normal and Low) for 12 consecutive days, where target value and range refers to package insert values.

	Normal Immunotrol^TM^		Low Immunotrol^TM^	
	CD4#	CD4%	CD4#	CD4%
**Number of values**	12	12	12	12
**Target value (package insert)**	**569±91**	**41.1±3.7**	**136±33**	**15.3±3.3**
**Target Range**	478–660	37.4–44.8	103–169	12.0–18.6
**Tested Range**	550–620	39.9–41.5	122–157	15.0–17.1
**Median ±Standard Deviation**	573.7±24.63	41.47±0.99	139.4±10.62	15.79±0.60
**95% Confidence Interval of Mean**	558–589.3	40.84–42.11	132.7–146.2	15.41–16.17
**%Coefficient of variation (precision)**	**4.29**	**2.41**	**7.62**	**3.82**

### Reproducibility

**Fig 3:**
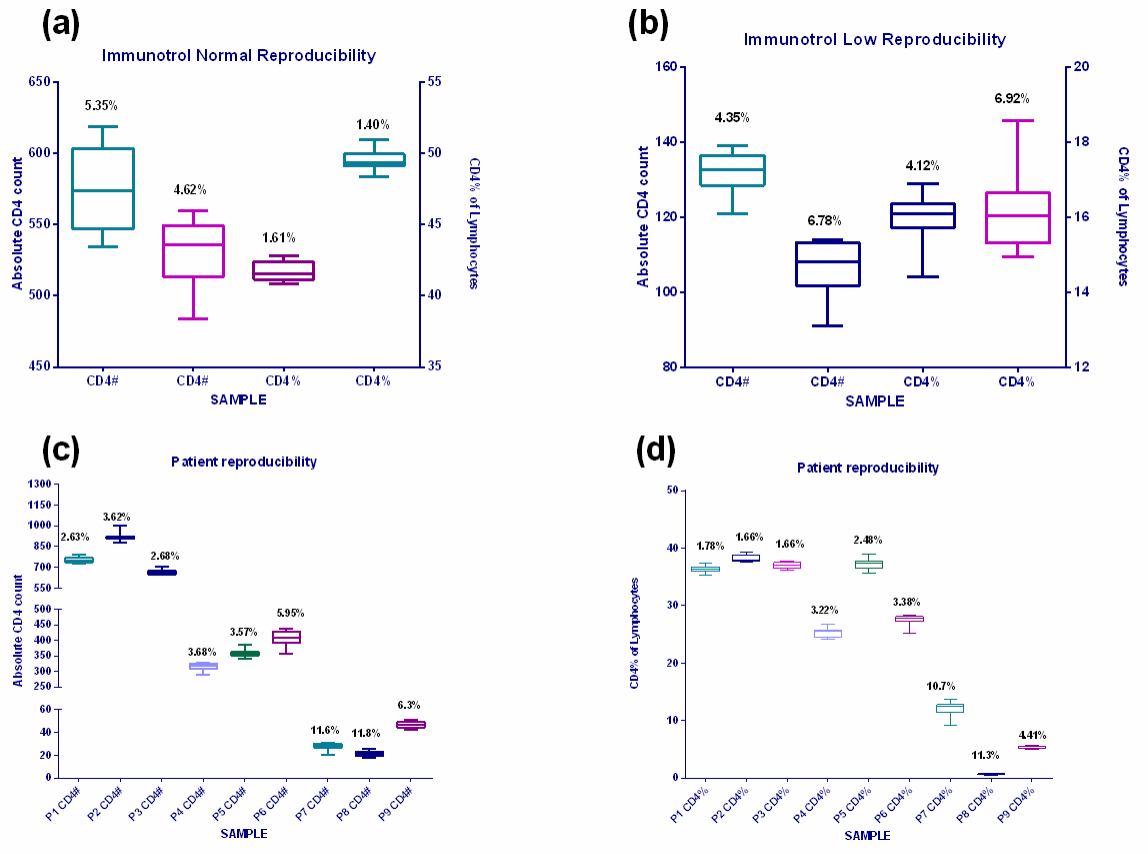
Reproducibility data on Aquios. Reproducibility data for absolute CD4 count and CD4% of lymphocytes compiled using internal quality control material, Immunotrol™ Normal (a) and Immunotrol™ Low (b). Reproducibility data of patient samples with a low, medium and high CD4 counts are represented for absolute CD4 counts (c) and CD4% of lymphocytes (d).

Reproducibility testing was undertaken with internal quality assessment material (Immunotrol^TM^ Normal and Low) (Fig 3A and 3B) and random patient samples with low, medium and high CD4 counts (Fig 3C and 3D). Reproducibility with two Immunotrol™ Normal samples (with target values of #CD4 569±91 and 550±165 cells/μl and CD4% of 41±3.7 and 50±9% respectively) showed within-target results with corresponding %CV values of <6%. Reproducibility values with Immunotrol^TM^ Low were within targets of 139±33 (tested mean of 132±5.9) and 114±57 (108±7.2) for #CD4 counts and 15.3±3.3 (18.9%±0.8) and 16±7 (18.1%±1.1) for CD4% values, with corresponding CV values <8% for both parameters.

Reproducibility (precision) results utilizing three patient samples per #CD4 category (low, medium and high) revealed %CV values for both parameters that were <4% at high CD4 counts (>500cells/μl) and <6% for medium counts (300–500 cells/μl), showing good reproducibility for both #CD4 counts and CD4%. The low values (<50cells/μl) had slightly elevated %CV’s (10–12%). The range of #CD4 counts with highest %CV values (of ˜11.6%) were in the range of 20–31 cells/μl (mean 27 vs. target of 38 cells/μl) and 18–26 cells/μl (mean 22 vs. target of 23 cells/μl) respectively. The corresponding CD4% values were 9.1–13.7% (with a mean of 12.1 vs. target 13.8%) and 0.6–0.8% (mean 0.7 vs. target of 0.66). The differences noted in #CD4 count and CD4% was not considered clinically significant (in that it would not change the clinical outcome or clinical intervention in patient care if detected in a clinical sample).

### External quality control (EQA) performance assessment

**Fig 4:**
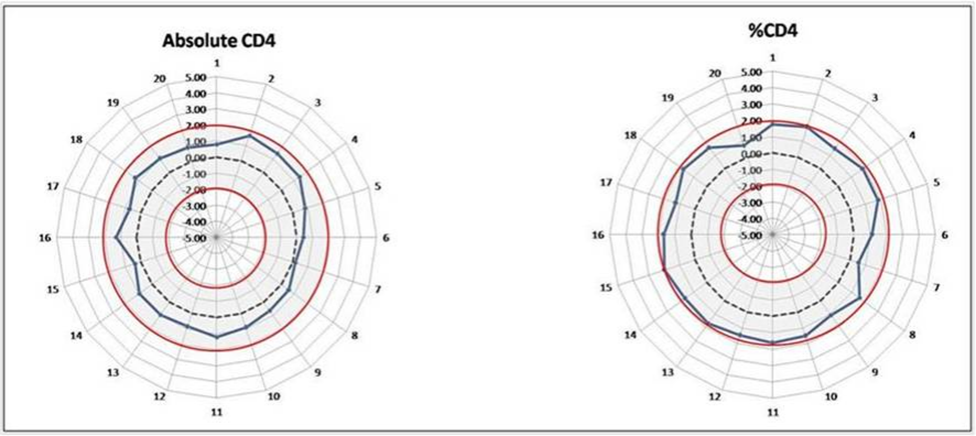
External quality assessment results. Radar graphs summarizing the results obtained with external quality assessment samples for absolute CD4 counts (left, including 10 trials, each with a low and a normal CD4 count on each respective trial) and CD4% of lymphocytes (right), both indicated by solid blue lines revealing the performance of the Aquios in relation to the respective 10 supplied retrospective trial pool means. The solid red lines indicate the acceptable 2 standard deviation range, while the interrupted black line represents the average overall performance (ideal) of the pooled results (at an SDI of zero).

Twenty EQA samples from 10 NHLS CD4 Proficiency Testing trials of EQA were analyzed on the Aquios instrument and an SDI calculated for each result based on the respective reported trimmed pool mean results of these trials. The EQA material included one medium/high CD4 count and one low CD4 count sample per trial. EQA panel samples tested on the Aquios platform delivered acceptable results with matched calculated SDI values for both absolute CD4 and CD4% parameters, i.e. all were shown to be within the calculated 2SD limit (Fig 4) of the reported trimmed pool means of the respective trials.

## Discussion

At the time when HIV treatment programs were first introduced into a relatively resource-limited South Africa (2004), the provision of wide-scale CD4 services proved to be challenging [30].Tests were expensive and skilled operators required to perform flow cytometry, were not readily available [31–33]. To provide cheaper and simpler services, alternative enumeration methods were initially adopted, i.e. manual CD4 counts or use of parameters such as the total lymphocyte count or CD4 percentage of lymphocytes as predictive indicators of the impact of HIV infection on the immune system [34–40]. The unfolding HIV pandemic and growing numbers of patients requiring care however provided an impetus for the rapid evolution of new generation flow cytometric CD4 T-cell enumeration systems to meet the needs of varying service delivery/test volumes. These varied from point-of-care approaches to high volume user-independent high-end systems that are now available to laboratories previously excluded from providing flow cytometry analysis for technical or financial reasons. Affordable, simpler gating strategies developed also played an important role in meeting local skills deficiency needs and costs constraints [12, 13, 41]. Consequently, current commercially available routine CD4 flow cytometry based testing systems now cover a wide spectrum of daily throughput, including both semi- and fully automated systems for medium to high daily (>500 samples/day) test volumes [42–45]. Development of point-of-care systems provided options for very low test volume settings (<15 samples per day) typically encountered in a clinical setting where placement of flow cytometry based testing systems is not appropriate [46–51].

The Aquios instrument evaluated here is one of the new generation standardized systems designed to meet the needs of cost-constrained settings, where technical skills may be lacking and service requirements demand the use of a more cost-efficient flow cytometric approach. The verification study presented here confirmed good overall agreement (accuracy of result reporting) of the new Aquios/PLG platform to the current NHLS predicate system currently used across a network of CD4 testing laboratories at various tiers of service need. There was consistent performance of the Aquios/PLG platform over time, across a wide range of absolute CD4 counts (2-1228cells/μl and median #CD4 of 365) with negligible intra-instrument between-platform bias (BA -40.5±44.0 cells/μl bias with LOA of 26.8 to 45.8 cells/μl). Good overall agreement and tight precision to predicate were reported (%Sim 94.83±5.39cells/μl and %SimCV of 5.69%). This makes it suitable for implementation across the South African national CD4 testing service as a replacement of the XL system without impacting clinical decision making of HIV patient care. Good precision of patient sample results as well as internal quality control products, were all confirmed by %CV values of <8%. System stability and reproducibility was also confirmed. These outcomes were further consolidated in an additional workflow study (n = 1885) to assess daily workflow capacity of the Aquios/PLG platform, simultaneously testing three systems over ten days. Good outcomes were also obtained with pediatric samples and intra-instrument comparisons (S1 File). Similar results have been published with the 4-color Aquios platform, i.e. slight under-estimation of absolute lymphocyte counts [52].

Although initially introduced as a 4-color platform (Tetrachrome™) [18], the system was modified and developed specifically to accommodate PLG/CD4 testing for the South African laboratory service and other countries using the PLG/CD4 methodology and replacement of redundant BC XL cytometers currently in use in these countries. In the South African network, majority of CD4 testing is performed in clinical pathology or hematology laboratories where technicians and technologists are required to multitask and perform all CD4 testing in addition to the other routine clinical pathology or hematology services offered in these sites. In this context, the fully automated on-board sample preparation incubation and analysis of the new Aquios is anticipated to improve the workflow and consistency of quality during testing across the South African network; no manual steps are required in the preparation of samples and on-board processing enables less qualified persons to properly operate the instruments without necessarily having flow cytometry background/training or skills in flow cytometry. The barcode scanning system also ensures that the correct reagents are used within their expiry dates and data about reagent usage, linked to each test result, is collected via the laboratory interfaced management system (LIMS) for national monitoring and collation of reagent use and lot numbers. Locked-down software further provides operator-independent quality and standardized testing by preventing inadvertent or intentional deleting or changing of protocols by the operator, vital for standardized use of protocols across a networked service. Training is also easier when fixed protocols are used. On-board quality assessment monitoring of parameters provides an additional advantage; quality management parameters can be also downloaded through the LIMS interface for collation of individual instrument and laboratory performance centrally. Centralized data collation further provides for remote, coordinated proactive monitoring of the quality of testing and instrument operation at all sites, valuable for assessing downtime and other operational risks in a national service. The advantages of this system thus favor routine testing laboratories where only one or two test sets are commonly used (PLG/CD4 and/or Tetrachrome 4-color). The closed system however is restrictive for research purposes where operators need to be able to freely create/develop new test protocols.

Workflow analysis indicated a daily 8-hour throughput of around 120 samples. The autoloader can take up to eight cassettes with five samples each, with the first result ready after approximately 20 minutes and thereafter every 2 minutes. Although this capacity is slightly less than the XL cytometers platform, it is foreseen that laboratories will be able to cope with their daily testing demands by changing their workflow. This will save on hands-on time, allowing laboratory staff to multitask/rotate in the context of clinical pathology services (including microbiology, chemistry and hematology) and consolidating staff time to enable the rollout of related services in the same testing facilities. The provision of reflexed screening of early opportunistic HIV co-infections such as Cryptococcal meningitis [53, 54] with Cryptococcal antigen (CrAg) testing of all CD4 samples with counts less than 100 cells/μl is one such example. For the implementation of this national testing program no additional staff were employed in any of the CD4 laboratories, i.e. fully integrated into routine CD4 testing [55, 56]. Time saving results were recently reported in a comparative study between the 4-color Aquios and the Beckton Dickinson FACSCanto II that assessed the impact of the new platform on laboratory turn-around-time [57]. With the Aquios capacity of 120 samples per day and operator-independent features reported here, the system is ideally suited for Tier-3 and lower-end level laboratories [58] with <200 test samples per day (2 Aquios systems). Furthermore, it can provide a means to extend laboratory services to non-CD4 testing laboratories where a need for better service provision is indicated [12, 58, 59]. In the South African context, we anticipate the biggest impact of the Aquios to be on both quality and service delivery (improved turn-around time) and increased decentralized service in smaller laboratories previously utilizing existing staff. Lastly, the auto-gating feature worked well, with <2% of samples needing operator-initiated re-gating, making standardization of result reporting in a network of testing facilities easier.

Data presented here indicated an overall consistent, slight under-estimation (~6%) of the absolute CD4 count compared to the MPL/CellMek PLG predicate method/platform, and excellent agreement of CD4 percentage of lymphocyte values. Similar results of slight under-estimation of absolute lymphocyte counts were reported in the validation of the 4-color Aquios system against the Beckman Coulter FC500 [52]. The under-estimation of absolute CD4 counts generated on true volumetric systems versus counts vs. single platform bead-based systems has been previously reported [60], including systems such as the Sysmex Cyflow (previously Partec) [61]. In a national network, notice to attending clinicians concerning a precise slight downward bias can provide for a once-off adjustment [11] for a patient’s CD4 count if the difference to the predicate result is consistent and precise as demonstrated on the Aquios (if the CD4 count was used to monitor response to treatment). Misclassification around pre-determined treatment threshold cut-offs are only significant if applied to patient care. These include (i) reflexed CrAg testing at a CD4<100cells/μl, (ii) ART initiation where either the 200, 350 or 500 cells/μl treatment thresholds are still followed, or (iii) fast tracking of patients onto therapy with low CD4 baseline counts, i.e. in South Africa, a CD4<200cells/μl is applied [62, 63]. A system that under-estimates CD4 counts would qualify more patients for treatment. However, the use of misclassification analyses in the prevailing context of the international trend towards doing away with the need for CD4 counts to define eligibility for antiretroviral treatment (ART) [9, 15, 28, 29, 64–66], are irrelevant if a universal test and treat (UTT) approach is applied. The only relevant misclassification level, if the WHO guideline [62] to screen for cryptococcal disease or other opportunistic infection is applied, is thus at the CD4 ≤100cells/μl level. This study indicated that the misclassification of patients at the 100cells/μl using the Aquios/PLG platform was acceptable at very low levels i.e. a 2.1% downward (in favor of screening) and 2.5% upward misclassification where patients would miss the opportunity for CrAg screening.

Lastly, in view of the recent WHO guideline recommendations and an international growing trend to apply UTT for all HIV+ patients, it is important to re-assess the future role of CD4 enumeration in the management of HIV+ patients accessing antiretroviral treatment (ART). Although the 2016 WHO guidelines support UTT [62] and do away with CD4 counts as an indicator of eligibility to start ART, a (miss)perception has arisen that CD4 counts are no longer integral to care of HIV+ patients accessing ART [54, 63, 67]. This underestimates the role of CD4 in determining the level of immunosuppression, i.e. how sick a patient is, especially those who present late for care (with very low CD4 counts). This is especially true in the context of countries with a very high patient burden with advanced HIV disease like South Africa [7]. It is thus important to emphasize that CD4 enumeration remains an important parameter [54, 63, 67] to assess immune status of patients at baseline before ART initiation, and/or for identifying risk and/or screening for opportunistic co-infections such as Cryptococcal disease [53, 54].

## Conclusion

The Aquios/PLG CD4 testing platform showed clinically acceptable result reporting, with good system stability and reproducibility. In the context of smaller clinical laboratories providing clinical pathology services in a tiered service delivery model and especially, lower-end tiers testing 120 or less CD4 samples per day, the system provides a pragmatic, practical and standardized solution for CD4 service implementation across a national network. The ease of use and accessibility of the small foot-print system enabled training of all levels of staff/ CD4 personnel with no prior flow Cytometry or CD4 testing background as the system is operator-independent, requires less hands-on time to enable multitasking in a clinical pathology setting, freeing staff to do additional laboratory tasks, as well as providing a system with on-board quality control with remote centralized monitoring to enable proactive system maintenance and large-scale continuous service delivery.

## Disclaimer

The views expressed here are those of the authors and not necessarily that of their sponsor and employer, the South African National Health Laboratory Service or the University of the Witwatersrand, Faculty of Health Sciences.

## Supporting information

S1 File Additional verification results. Summary of additional verification parameters assessed on the Aquios CL PLG platform for the purpose of conforming to in-house verification protocol requirements of new instruments prior to implementation.
(DOCX)

S1 Table Raw data for comparative data (n = 205). Summary of raw data used for analysis of comparative dataset (n = 205) for the purpose of this paper.
(XLSX)

S2 Table Raw data for comparative data (n = 1885). Summary of raw data used for workflow analysis and comparison for the purpose of this paper.
(XLSX)
