# Supplementary material for: Performance verification of the new fully automated Aquios flow cytometer PanLeucogate (PLG) platform for CD4-T-lymphocyte enumeration in South Africa
Source: PLoS One. 2017 Nov 3;12(11):e0187456. doi: 10.1371/journal.pone.0187456 (PMC5669480; doi:10.1371/journal.pone.0187456)
Supplement: S1 File — Summary of additional verification parameters assessed on the Aquios CL PLG platform for the purpose of conforming to in-house verification protocol requirements of new instruments prior to implementation. (DOCX) [file pone.0187456.s001.docx]

**Supplementary data:**

**Methods:**

Additional daily internal quality assessment measures included background count and carry-over and weekly reproducibility to comply with current CD4 enumeration practices in the NHLS. Additional performance qualification (PQ) measures included inter-instrument comparison, impact of sample age on accurate absolute CD4 (#CD4) and accuracy of reporting paediatric results.

**Results:**

**Background count and carry over**

Background counts were consistently <10 events/µl. Carry over was negligible at <1% over the testing period.

**Inter-instrument and inter-platform variability:**

Two Aquios instruments were set up in the testing laboratory where a subset of 50 random patient samples were analysed on both systems within 24 hours of a resulted CD4 by predicate PLG/MPL CellMek method in the reference CD4 laboratory. A range of CD4 counts were included, i.e. 2-1658 cells/µl with a mean of 424 cells/µl. %Similarity analyses was done with the predicate as test vs. Aquios 1 or Aquios 2 and additionally Aquios 1 vs. Aquios 2 (Supplement Fig 1). The %similarity for #CD4 of Aquios platforms 1 and 2 vs. the predicate MPL was 96.6±3.9 and 95.5±3.9% respectively with CV’s <4%. For CD4% the similarity between the predicate and either Aquios platform was close to 100% with CV’s<3%. Comparison between the two Aquios systems showed similar results with tight CV values for both parameters tested (Fig 3). Bland-Altman analyses confirmed the good overall comparison between instruments/platforms with a bias of -30.7±38.3 (95% LOA -105 to 44) for Aquios 1 vs. MPL (intra-platform); -43.7±39.6 (95% LOA -121 to 34) for Aquios 2 vs. MPL (intra-platform) and -12.9±27 (95% LOA -66 to 40) for Aquios 1 vs. Aquios 2 (inter-instrument). Bias values for CD4% was negligible for all three comparisons (difference <1% with 95% LOA from -3 to 2.3).


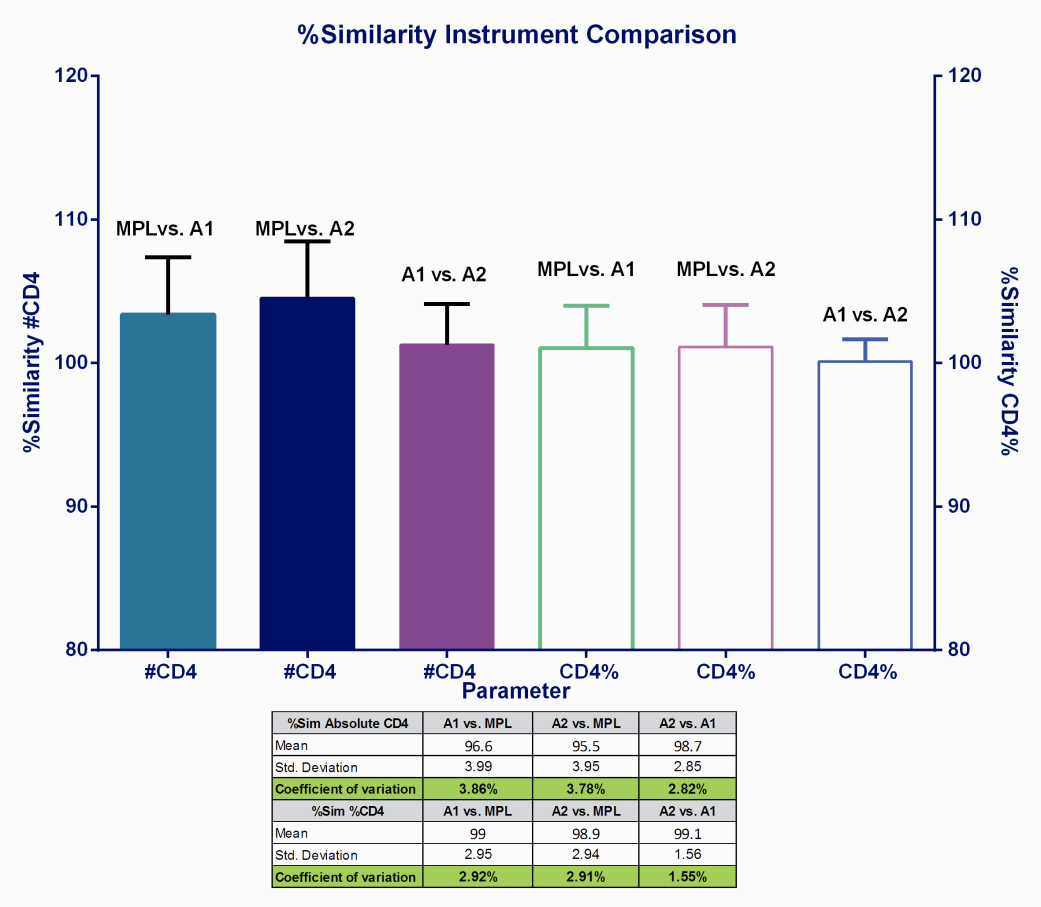


**S Fig 1: Inter-instrument comparison of absolute CD4 count and CD4% of lymphocyte reporting, using the %similarity.**

**Impact of sample age on accuracy:**

Ten random patient CD4 samples with confirmed results collected on day one of analyses were re-tested daily for five consecutive days and results compared to the original predicate values reported. This analysis confirmed the slight under-estimation of absolute CD4 counts compared to the predicate PLG/MPL CellMek results (Day 1 vs. Target, Suppl Fig 1). However, over time, the loss of absolute CD4 counts was not statistically or clinically relevant and would not change the outcome of patient care.

The testing was taken well beyond (48 hours) the published (package insert) recommendation of testing within 72 hours (3 days) of venesection.


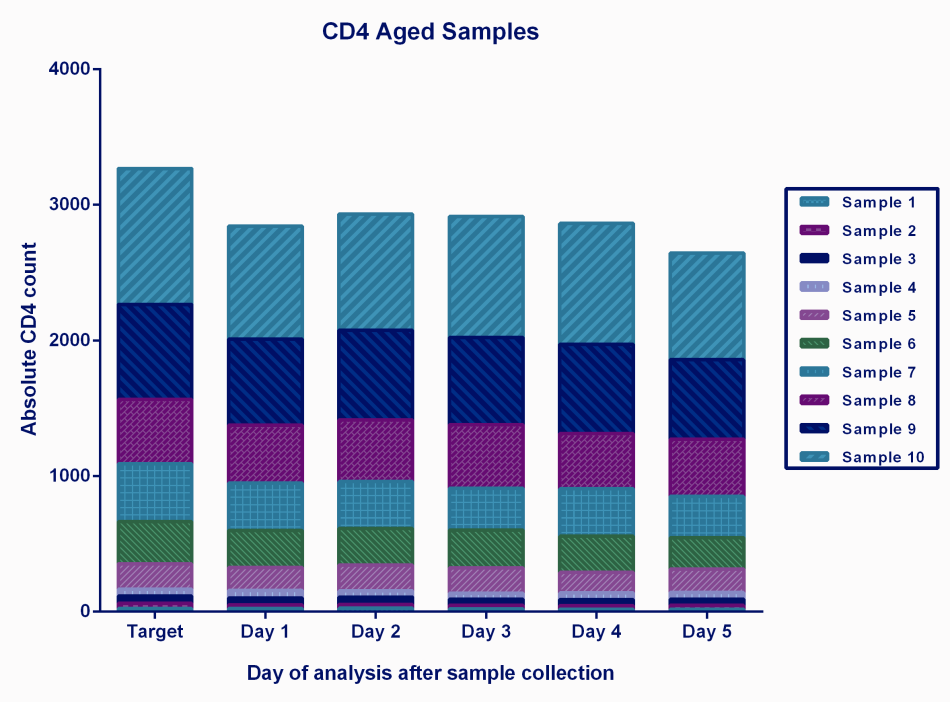


**S Fig 2: Impact of aged samples on accurate absolute CD4 count reporting by the Aquios compared to the predicate PLG/CD4.**

**Paediatric results comparison**

25 paediatric samples were tested with good overall comparison to the predicate method, with a slight under-estimation of absolute CD4 counts as seen in adult patient samples (S2 Table).%Similarity for absolute CD4 counts were 95.2±7% with a corresponding %CV of 7.4% and 99±3.2 for CD4% of lymphocytes with a %CV of 3.3%.
